# The Mental Health of Older People Living in Nursing Homes in Northern Portugal: A Cross-Sectional Study Protocol

**DOI:** 10.3390/nursrep15010024

**Published:** 2025-01-16

**Authors:** Cláudia Rodrigues, Sandra Carreira, Rui Novais, Fátima Braga, Silvana Martins, Odete Araújo

**Affiliations:** 1 School of Nursing, University of Minho, 4710-057 Braga, Portugal; rnovais@ese.uminho.pt (R.N.); fbraga@ese.uminho.pt (F.B.); odete.araujo@ese.uminho.pt (O.A.); 2 Nursing Research Centre, University of Minho, 4710-057 Braga, Portugal; up201202120@up.pt; 3 Instituto de Ciências Biomédicas Abel Salazar (ICBAS), University of Porto, 4050-313 Porto, Portugal; 4 Health Sciences Research Unit: Nursing (UICISA: E), Nursing School of Coimbra (ESEnfC), 3045-043 Coimbra, Portugal; 5 ProChild CoLAB Against Poverty and Social Exclusion—Association, 4810-225 Guimarães, Portugal; silvana.martins@prochildcolab.pt

**Keywords:** mental health, aged, nursing homes, health of institutionalized elderly, frailty, institutionalization

## Abstract

**Background/Objectives**: In Portugal, evidence regarding the mental health of institutionalized older people is limited, leaving this area poorly described and the mental health needs of this population largely unknown. This research aims to describe the mental health of older persons residing in nursing homes in Northern Portugal. **Methods**: A cross-sectional study will be conducted. We estimate that 567 participants will be recruited through convenience sampling. Potential participants must live in nursing homes in Northern Portugal, be aged 65 years or older, and exhibit cognitive impairment at an initial or intermediate stage. Ten web survey questionnaires will be administered to the participants, including one sociodemographic and health questionnaire and nine mental health assessment instruments evaluating fear of falling; sleep quality; frailty; anxiety, depression, and stress; loneliness and social isolation; risk of acute confusion; cognition; emotional literacy; and perceived hope. Data will be analyzed by employing descriptive, cluster, inferential, and bivariate analyses, with multiple regression models included. The study and the research protocol were submitted to and approved by the Ethics Committee of a major public university in Northern Portugal (CEICVS 007/2025). **Expected Results:** This is a pioneering study in Portugal, representing the first attempt to assess the mental health of older nursing home residents. Our study will enhance the understanding of the mental and multifactorial health needs of this population through a comprehensive description of their mental health, and sociodemographic and health characteristics.

## 1. Introduction

Extended longevity and the increase in average life expectancy have catalyzed population aging and driven a profound transformation in the demographic structure of contemporary societies. In 2023, older people (aged 65 years and over) constituted 21.3% of the European population, with Portugal and Italy presenting even higher proportions at 24% of their total population. In Europe, older persons exhibit a significantly high old-age dependency ratio (33.4% in 2023), while Portugal presents an even higher ratio of 38%. It is projected that by 2100, older people will comprise 32.5% of the European population and the old-age dependency ratio will expand to 59.7% [1].

Aging, rather than being synonymous with dependency, is a heterogeneous phenomenon that defines which conditions and contexts older people will experience in the final stages of their lives. It is widely recognized that older adults prefer to remain in their communities—a concept known as ‘aging in place’ [2]—but the increasing institutionalization of older people in nursing homes has proven to be an effective and appropriate response to their physical and mental health needs. In fact, with advancing age, older people look for environments that compensate for age-related changes, such as declines or losses in functional or cognitive capacity [3].

In Portugal, the year 2020 recorded a total of 2526 nursing homes with 99,234 institutionalized older people [4]. In 2022, 23,668 older people were residing in nursing homes in the Northern region of Portugal. From 2000 to 2022, nursing homes were the most expanded social response for older people, accounting for 78.4% of available services. In particular, this social response presented the highest average utilization rate, with 91.8% of available beds occupied in the continental territory [5].

The advanced age of older nursing home residents is often associated with a high prevalence of chronic health conditions, which leads to increased physical and cognitive frailty [6]. Socci et al. [7] conducted a study to assess the sociodemographic, functional, and psychosocial profile of older people residing in nursing homes across Europe, which supports the previous evidence. Their research shows that this population comprises individuals aged 80 years or older, mainly female, and often widowed with a small social network size and low levels of education. Additionally, older nursing home residents frequently present multiple chronic health conditions, experience functional limitations, and perceive their health status as fair to poor, alongside a low quality of life and moderate to low satisfaction with life.

In 2022, mental health disorders were reported as the third most prevalent issue, affecting 70.5% of the older people residing in nursing homes across Portugal [5]. However, current evidence regarding the health of institutionalized older people predominantly emphasizes their physical health profile and the impact of physical health conditions exacerbated by physical frailty, as highlighted by Socci et al. [7].

Frailty in older people is widely addressed in the literature as the consequence of losses in one or more domains of human functioning: physical, psychological, and social [8]. Physical frailty is characterized by a decline in physical capacity, particularly in mobility, endurance, muscle strength, and brain function [9]. Frailty is also a multidimensional phenomenon associated with significant psychological and social losses. The literature describes social frailty as the result of social isolation, along with a lack of meaningful interactions and adequate support. On the other hand, psychological frailty is defined as a decline in cognitive function, mood, and coping mechanisms, contributing to a state of psychological vulnerability in older people [8]. Thus, to effectively foster an environment that promotes successful aging, nursing homes must adopt a proactive and interdisciplinary approach and address both the physical and mental health needs of institutionalized older people.

The mental health of older nursing home residents has begun to be investigated, particularly the fear of falling [10], sleep disorders [11], anxiety and depression [12], neurocognitive impairment [13], loneliness [14], risk of acute confusion [15], and hopelessness [16].

A narrative review reported that the prevalence of delirium across institutionalized older persons varies widely, ranging from 1.4% to 70.3% [15]. In fact, the risk of acute confusion and fear of falling are major manifestations of physical frailty [9]. The fear of falling may increase dependency by decreasing confidence and mobility, and subsequently psychological and social frailty through progressively reduced participation in group psychosocial activities [17]. Moreover, a recent study indicated that severe activity restriction represents 60% of the total effect of fear of falling on depression across nursing home residents [10].

Sleep disturbances are prevalent among older nursing home residents, with approximately 95.8% of this population reporting a poor sleep quality [18]. Previous studies have also suggested an association between a poorer quality of life and poorer sleep quality and frailty [11,18].

A systematic review and meta-analysis suggested that 61% of institutionalized aged people may experience moderate loneliness, while 35% may suffer from severe loneliness [14]. Social isolation and loneliness enhance social frailty through the fragmentation of interpersonal relationships [19]. Loneliness is also recognized as a risk factor for depression and anxiety, and, consequently, contributes to psychological frailty. Thus, loneliness represents another risk factor for physical, psychological, and social frailty, and the evidence indicates the existence of bidirectional associations between loneliness, particularly social loneliness, and frailty [20].

Recent research indicates that anxiety is highly prevalent among older people residing in nursing homes and is often associated with depression and cognitive impairment [21]. A systematic review and meta-analysis reported that the pooled prevalence of mild cognitive impairment in this demographic is 21.2% [13]. Furthermore, an increase in dementia cases is anticipated [22]. The study by Šare et al. [12] also suggests an association between advanced age and depression, as well as with gender and anxiety. Psychological frailty often comes with a sense of hopelessness [16], which has inspired new studies to assess the concepts of emotional literacy [23] and perceived hope [24].

In Portugal, evidence regarding the sociodemographic and health profile of institutionalized older people is limited. Specifically, the mental health of older nursing home residents remains underexplored, leaving the mental health profile of this population poorly characterized and their mental health needs largely unknown. Based on this background, our research is guided by the following research question: What is the association between mental health and the sociodemographic characteristics of older people residing in nursing homes? Furthermore, this study aims to describe the mental health of older people living in nursing homes in Northern Portugal. The secondary objectives of this study are (i) describing the sociodemographic and health profile of older people living in nursing homes in Northern Portugal; and (ii) analyzing the associations between their mental health profile and the sociodemographic and health characteristics of this population.

## 2. Methods

### 2.1. Design and Setting

This is a correlational descriptive study, to be conducted with a cross-sectional design within a quantitative approach. It is expected to be conducted in multiple nursing homes across three districts of Northern Portugal: *Braga*, *Porto*, and *Viana do Castelo.* Nursing homes are specialized facilities that provide multidisciplinary healthcare to older people, who reside there permanently and have significant healthcare needs or lack social support.

### 2.2. Participants

Through the use of a statistical formula, the required sample size has been estimated at 567 participants, with a 95% confidence interval [25]. Older people from various nursing homes will be recruited through a non-probability convenience sampling technique.

Older people will be eligible to participate in this study if they meet the following inclusion criteria: (i) aged 65 years or older, (ii) living in a nursing home, and (iii) exhibit cognitive impairment at an initial or intermediate stage. The participants will be excluded if (i) they have cognitive impairment at an advanced stage; and (ii) they are unable to engage in verbal communication.

## 3. Materials and Equipment

The instruments used in this study comprise a sociodemographic and health questionnaire and nine mental health assessment instruments that have been validated, rigorously tested, and culturally adapted for the Portuguese population.

### 3.1. Sociodemographic and Health Questionnaire

The questionnaire evaluates sociodemographic and health characteristics through a set of variables, including age, gender, marital status, and educational level. It further assesses the elements, frequency, and satisfaction levels associated with social networks, the duration of institutionalization, chronic health conditions, and functional limitations.

For this study, educational level is defined according to the National Qualifications System of Portugal. The social network will be evaluated based on its elements, frequency of contacts, and the satisfaction levels reported by older people. In particular, the satisfaction levels associated with social networks will be measured using a self-report scale of 0 to 10, where higher scores correspond to greater levels of satisfaction [26]. Regarding functional limitations, participants will be asked about their ability to perform six basic activities of daily living: dressing, walking across a room, bathing, eating, getting in or out of bed, and using the toilet [27]. A brief description of each variable is presented in Table 1.

### 3.2. Psychometric Scales

The mental health profile of older people living in nursing homes will be determined through the application of nine psychometric scales. Each scale evaluates distinct constructs: fear of falling [28]; sleep quality [29]; frailty [30]; anxiety, depression, and stress [31]; loneliness and social isolation [32]; risk for acute confusion [33]; cognition [34]; emotional literacy [35]; and perceived hope [24]. A summary description of each instrument is provided in Table 2.

## 4. Detailed Procedure

### 4.1. Data Collection

For this study, research teams have been established to conduct data collection. To ensure procedural consistency and reduce potential bias, data collectors will receive prior training and specific guidance on the administration of the web survey questionnaires.

**Figure 1:**
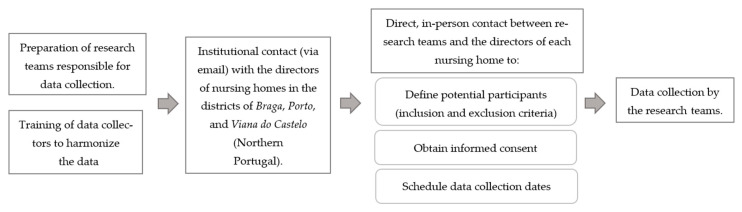
Procedure before data collection.

Data collection will be conducted according to prior agreements established between the directors of each participating nursing home and the research teams. Figure 1 presents a detailed description of the procedure before data collection.

Given the extensive range of instruments required, a comprehensive data collection tool was developed in EUSurvey, comprising ten web survey questionnaires: one sociodemographic and health questionnaire and nine mental health assessment instruments. For data collection, electronic surveys have been chosen due to the efficiency of data entry and analysis, low administration costs, and flexibility in mitigating invalid responses and minimizing missing data [36].

Data collection will be carried out in phases to ensure the participants’ health statuses and availability are respected. It is estimated that participants will complete the web survey questionnaires in approximately 60 min. During this time, research teams will be available to support participants and assist with the completion of the questionnaires. All web survey questionnaires will be assigned with an alphanumeric code to preserve anonymity and to document the sequence of the data collection in each participating nursing home.

### 4.2. Data Analysis

The data collected will be analyzed using the Software Statistical Package for Social Science (SPSS Inc., Chicago, IL, USA), version 28.0.

Descriptive statistical analyses will be conducted to characterize the sociodemographic, health, and mental health profiles of older people living in nursing homes in Northern Portugal. Categorical variables will be summarized using relative frequencies. Continuous and discrete variables will be described through measures of central tendency (mean and median) and relative dispersion indices (standard deviation).

Cluster analysis will be executed to create profiles according to the sociodemographic and health characteristics of the participants. Inferential analysis will be performed using parametric or non-parametric tests, according to the normality and the distribution of the quantitative variables. A significance level of *p* < 0.05 was established for all analyses [37].

Bivariate analysis will be performed to describe associations between the mental health profile and sociodemographic and health profiles, as well as amongst variables within the participants’ mental health profile. To evaluate these relationships, the Pearson or Spearman correlation will be applied. Considering the types of variables, we will conduct a chi-square test or mean comparison tests. Based on the results of previous analysis, multiple regression models will be constructed for each mental health variable to better understand which sociodemographic and health characteristics are predictive for each variable.

### 4.3. Ethical Considerations

This study and research protocol were approved by the Ethics Committee of a major public university in Northern Portugal (CEICVS 007/2025).

The research teams will provide all eligible older people with detailed information regarding the aim, objectives, and purpose of this study, as well as the voluntary participation conditions, data confidentiality, and the option to dropout without personal harm or further consequences. Subsequently, a written informed consent will be presented to all participants in person, and their signature will be requested.

## 5. Expected Results

This is a pioneering study in Portugal and the first attempt to assess the mental health of older people in nursing homes through an integrated and comprehensive set of psychological, physical, and sociodemographic variables defined following the most recent evidence. The inclusion of a diverse sample and using a multidimensional methodology in our study will enable a comprehensive description of the mental health of this population, along with its sociodemographic and health characteristics. Thus, we will acquire a comprehensive understanding of the multifactorial health needs of institutionalized older people and their influence on mental health.

An important function of this study is to raise awareness of age-related changes in mental health within nursing homes. The results will demonstrate whether sociodemographic and health variables directly influence the mental health profile of institutionalized older people, thus highlighting the biopsychosocial perspective on the frailty of this population [38]. Therefore, we will examine the relationships among the mental health profile variables, which will be used in an integrated manner for the first time in this study.

This area remains underexplored in the current literature, and the findings from this study will enhance our understanding of the mental health of older people living in this social setting. Moreover, it will provide insights into the factors contributing to the third most prevalent type of problem among older people living in nursing homes across Portugal: mental health disorders [5].

Our study has the potential to make significant contributions to the scientific community and healthcare professionals working in institutional settings, as it will provide new insights into priority mental health interventions for older people living in nursing homes. This study will also provide valuable information for the development of new strategies, intervention programs, guidelines, and health policies intended to improve the healthcare provided by multidisciplinary teams in nursing homes and the distribution of health resources, as well as offer direction on how to incorporate new healthcare professionals as a measure to better address the multifactorial healthcare needs of this population.

### Limitations

This study has some expected limitations that should be acknowledged, particularly related to the data collection. Institutionalized older people are more likely to present neurodegenerative or cerebrovascular diseases associated with advanced age, which may result in challenges in achieving the required sample size. Another difficulty, also related to advanced age, is the potential loss of sample participants due to mortality or attrition. The limited number of trained staff in nursing homes may also impact the quality of data collected. Moreover, the research teams may introduce potential bias as variations in data collection among the data collectors could occur. To address this potential limitation, data collectors will be provided with prior training and detailed instructions on the administration of web survey questionnaires, ensuring a standardized and consistent data collection process.

## Figures and Tables

**Table 1:** Sociodemographic and health questionnaire variables.

Variable	Method of Measurement	Operational Definition
Age	Clinical Data/ Patient Report	Years
Gender	Patient Report	Female versus male
Marital status	Clinical Data/ Patient Report	Single, married, divorced, or widowed
Educational level	Clinical Data/ Patient Report	No schooling, attended school without completing any educational level, 1st cycle of primary education, 2nd cycle of primary education, 3rd cycle of primary education, upper-secondary education, upper-secondary education obtained via dual certification, post-secondary education, bachelor’s degree, master’s degree, or doctorate
Social network elements	Patient Report	I lack social support from others, sister(s)/brother(s), daughter(s)/son(s), granddaughter(s)/grandson(s), daughter-in-law/son-in-law, friend(s), neighbors, and others
Frequency of contacts	Clinical Data/ Patient Report	Daily, weekly, several times per week, biweekly, monthly, and other
Satisfaction levels associated with social network	Patient Report	0–10
Duration of institutionalization	Clinical Data/ Patient Report	<1 year, 1–5 years, 6–10 years, 11–15 years, and ≥16 years
Chronic health conditions	Clinical Data/ Patient Report	Yes or no Then, enumerate them
Functional limitations	Clinical Data/ Patient Report	Dressing, walking across a room, bathing, eating, getting in or out of bed, and using the toilet

**Table 2:** The psychometric scales used to assess the mental health profile.

Variable	Psychometric Scale	Authors	Items	Dimension(s)	Reliability	Rating Scale	Global Classification
Fear of falling	FEIS-I_(P)_: European Portuguese version of the Falls Efficacy Scale—International	Figueiredo and Santos (2017) [28]	16	1	α = 0.978	4-point Likert scale (from 1 = not all concerned to 4 = very concerned)	Maximum: 64 (greater fear of falling)
Sleep quality	PSQI-PT: Portuguese version of the Pittsburgh Sleep Quality Index	Del Rio João et al. (2017) [29]	24	7 Subjective sleep quality, sleep latency, sleep duration, habitual sleep efficiency, use of sleeping medication, and daytime dysfunction	α = 0.70	4-point scale (from 0 to 3)	Cut-off point: >5 (major difficulties in ≥2 dimensions or moderate difficulties in >3 dimensions)
Frailty	TFI: Portuguese version of the Tilburg Frailty Indicator	Coelho et al. (2015) [30]	25 2 subscales: Part A—10 items Part B—15 items	2 Determinants of frailty and total frailty (physical, psychological, social)	KR-20 = 0.78	11 items: dichotomous scale (yes/no) 4 items: trichotomous scale (no/sometimes/yes) Rated from 0 to 1	Cut-off point: 6 (frailty)
Anxiety, depression, and stress	EADS-21: Portuguese version of the Depression Anxiety Stress Scales	Pais-Ribeiro et al. (2004) [31]	21 3 subscales, each with 7 items	3 Anxiety ^1^, depression ^2^, and stress ^3^	α = 0.74 ^1^; 0.85 ^2^; 0.81 ^3^	4-point Likert scale: (from 0 = did not apply to me at all to 3 = applied to me most of the time)	Maximum: 21 for each subscale (the most negative affective states are identified)
Loneliness and social isolation	Portuguese version of the UCLA-Loneliness Scale	Pocinho et al. (2010) [32]	16	2 Social isolation and affinities	α = 0.905	4-point Likert scale: (from 1 = never to 4 = always)	Cut-off point: >32 (presence of negative feelings of loneliness)
Risk of acute confusion	*Escala de avaliação do risco de confusão aguda*	Vieira (2022) [33]	13	1	*	Dichotomous scale (present/absent)	
Cognition	Portuguese version of the Montreal Cognitive Assessment (MoCA)	Freitas et al. (2010) [34]	11	8 Attention, concentration, memory, working memory, executive function, language, visuoconstructional skills, and temporal and spatial orientation	α = 0.94	Each correct response is rated as 1 point	Maximum: 30 (better cognitive function)
Emotional literacy	Portuguese version of the Emotional Literacy Skills Scale	Sousa et al. (2022) [35]	31	5 Motivation, empathy, self-regulation, emotional awareness, social skills	α = 0.755	5-point Likert scale: (from 1 = never true to 5 = almost always true)	3.41–5.00: strong emotional literacy 2.61–3.40: need for attention and promotion of emotional literacy <2.60: priority intervention in emotional literacy
Perceived hope	PHS: Portuguese version of the Perceived Hope Scale	Marujo et al. (2021) [24]	6	1	α = 0.88–0.89	6-point Likert scale: (from 0 = strongly disagree to 5 = strongly agree)	Maximum: 30 (high level of perceived hope)

Note. * The psychometric properties of this scale will be validated within the scope of this study. α: Cronbach’s alpha, KR-20: Kuder–Richardson Formula 20.

## Data Availability

Data will be available from the author upon reasonable request.

## Public Involvement Statement

No public involvement in any aspect of this research.

## Guidelines and Standards Statement

This manuscript was drafted in adherence to the EQUATOR Network (Enhancing the QUAlity and Transparency Of health Research) and following the SPIRIT 2013 statement (Standard Protocol Items: Recommendations for Interventional Trials).

## References

[1] Eurostat Statistics Explained. https://ec.europa.eu/eurostat/statistics-explained/index.php?title=Population_structure_and_ageing.

[2] Melchiorre M.G., D’Amen B., Quattrini S., Lamura G., Socci M. (2022). Caring for Frail Older People Living Alone in Italy: Future Housing Solutions and Responsibilities of Family and Public Services, a Qualitative Study. Int. J. Environ. Res. Public Health.

[3] European Commission: Directorate-General for Employment, Social Affairs and Inclusion (2021). Long-Term Care Report: Trends, Challenges, and Opportunities in an Aging Society.

[4] Serviço Nacional de Saúde. https://www.sns.gov.pt/noticias/2020/08/12/estruturas-residenciais-para-idosos/.

[5] Gabinete de Estratégia e Planeamento. https://www.cartasocial.pt/destaques/-/asset_publisher/ZxGHxe4w3QOc/content/relatorio-2022-ja-disponivel.

[6] Kojima G. (2015). Prevalence of Frailty in Nursing Homes: A Systematic Review and Meta-Analysis. J. Am. Med. Dir. Assoc..

[7] Socci M., Di Rosa M., D’Amen B., Melchiorre M.G. (2023). Functional and Psychosocial Profile of Older People Living in Nursing Homes: Findings from the European Survey of Health, Ageing, and Retirement in Europe (SHARE). Healthcare.

[8] Gobbens R.J.J., Uchmanowicz I. (2023). Frailty Viewed From a Nursing Perspective. SAGE Open Nurs..

[9] Clegg A., Young J., Iliffe S., Rikkert M.O., Rockwood K. (2013). Frailty in elderly people. Lancet.

[10] Xu D., Wang Y., Zhu S., Zhao M., Wang K. (2024). Relationship between fear of falling and quality of life in nursing home residents: The role of activity restriction. Geriatr. Nurs..

[11] Lorber M., Kmetec S., Davey A., Mlinar Reljić N., Fekonja Z., Kegl B. (2023). Associations between Sleep Quality, Frailty, and Quality of Life among Older Adults in Community and Nursing Home Settings. Int. J. Environ. Res. Public Health.

[12] Šare S., Ljubičić M., Gusar I., Čanović S., Konjevoda S. (2021). Self-Esteem, Anxiety, and Depression in Older People in Nursing Homes. Healthcare.

[13] Chen P., Cai H., Bai W., Su Z., Tang Y.L., Ungvari G.S., Ng C.H., Zhang Q., Xiang Y.T. (2023). Global prevalence of mild cognitive impairment among older adults living in nursing homes: A meta-analysis and systematic review of epidemiological surveys. Transl. Psychiatry.

[14] Gardiner C., Laud P., Heaton T., Gott M. (2020). What is the prevalence of loneliness amongst older people living in residential and nursing care homes? A systematic review and meta-analysis. Age Ageing.

[15] Komici K., Guerra G., Addona F., Fantini C. (2022). Delirium in Nursing Home Residents: A Narrative Review. Healthcare.

[16] Drageset J., Haugan G., Tranvag O. (2017). Crucial aspects promoting meaning and purpose in life: Perceptions of nursing home residents. BMC Geriatr..

[17] Birhanie G., Melese H., Solomon G., Fissha B., Teferi M. (2021). Fear of falling and associated factors among older people living in Bahir Dar City, Amhara, Ethiopia- a cross-sectional study. BMC Geriatr..

[18] Kumar S., Wong P.S., Hasan S.S., Kairuz T. (2019). The relationship between sleep quality, inappropriate medication use and frailty among older adults in aged care homes in Malaysia. PLoS ONE.

[19] Davies K., Maharani A., Chandola T., Todd C., Pendleton N. (2021). The longitudinal relationship between loneliness, social isolation, and frailty in older adults in England: A prospective analysis. Lancet Healthy Longev.

[20] Ye L., Bally E., Korenhof S.A., Fierloos I., Alhambra Borras T., Clough G., Raat H., van Grieken A. (2024). The association between loneliness and frailty among community-dwelling older adults in five European countries: A longitudinal study. Age Ageing.

[21] Creighton A.S., Davison T.E., Kissane D.W. (2019). The Factors Associated With Anxiety Symptom Severity in Older Adults Living in Nursing Homes and Other Residential Aged Care Facilities. J. Aging Health.

[22] GBD 2019 Dementia Forecasting Collaborators (2022). Estimation of the global prevalence of dementia in 2019 and forecasted prevalence in 2050: An analysis for the Global Burden of Disease Study 2019. Lancet Public Health.

[23] Alemdar M., Anilan H. (2020). The Development and Validation of the Emotional Literacy Skills Scale. Int. J. Contemp. Educ. Res..

[24] Marujo H.Á., Velez M.J., Gonçalves S.P., Neto L.M., Krafft A.M., Casais M. (2021). The value of hope: Validation of the perceived hope scale in the Portuguese population. Curr. Psychol..

[25] Dell R.B., Holleran S., Ramakrishnan R. (2002). Sample size determination. ILAR J..

[26] Litwin H., Stoeckel K.J., Schwartz E. (2015). Social networks and mental health among older Europeans: Are there age effects?. Eur. J. Ageing.

[27] Pavlidis G., Hansen T., Motel-Klingebiel A., Aartsen M. (2022). Network and solitude satisfaction as modifiers of disadvantages in the quality of life of older persons who are challenged by exclusion from social relations: A gender stratified analysis. Appl. Res. Qual. Life.

[28] Figueiredo D., Santos S. (2017). Cross-cultural validation of the Falls Efficacy Scale-International (FES-I) in Portuguese community-dwelling older adults. Arch. Gerontol. Geriatr..

[29] Del Rio João K.A., Becker N.B., Neves Jesus S., Isabel Santos Martins R. (2017). Validation of the Portuguese version of the Pittsburgh Sleep Quality Index (PSQI-PT). Psychiatry Res..

[30] Coelho T., Santos R., Paúl C., Gobbens R.J., Fernandes L. (2015). Portuguese version of the Tilburg Frailty Indicator: Transcultural adaptation and psychometric validation. Geriatr. Gerontol. Int..

[31] Pais-Ribeiro J.L., Honrado A., Leal I. (2004). Contribuição para o estudo da adaptação portuguesa das Escalas de Ansiedade, Depressão e Stress (EADS) de 21 itens de Lovibond e Lovibond. Psicologia, Saúde & Doenças. Soc. Port. Psicol. Saúde.

[32] Pocinho M., Farate C., Dias C.A. (2010). Validação Psicométrica da Escala UCLA-Loneliness para Idosos Portugueses. Interações Soc. Novas Mod..

[33] Vieira T. (2022). Confusão Aguda em Pessoas Mais Velhas: Contributos Para a Avaliação do Risco. Masters’ Thesis.

[34] Freitas S., Simões M.R., Martins C., Vilar P.M. (2010). Estudos de Adaptação do Montreal Cognitive Assessment (MoCA) para a População Portuguesa. Aval. Psicol..

[35] Sousa L. Competência Emocional em Enfermeiros: Contributos da Inteligência Artificial. Proceedings of the XII Congresso Internacional d’A Sociedade Portuguesa de Enfermagem de Saúde Mental.

[36] Evans J.R., Mathur A. (2018). The value of online surveys: A look back and a look ahead. Internet Res..

[37] Marôco J. (2010). Análise Estatística com o SPSS.

[38] Gobbens R.J., Luijkx K.G., Wijnen-Sponselee M.T., Schols J.M. (2010). Towards an integral conceptual model of frailty. J. Nutr. Health Aging.

